# Neural Crest Stem Cells from Dental Tissues: A New Hope for Dental and Neural Regeneration

**DOI:** 10.1155/2012/103503

**Published:** 2012-10-04

**Authors:** Gaskon Ibarretxe, Olatz Crende, Maitane Aurrekoetxea, Victoria García-Murga, Javier Etxaniz, Fernando Unda

**Affiliations:** Department of Cell Biology and Histology, Faculty of Medicine and Dentistry, University of the Basque Country (UPV/EHU), 48940 Bizkaia, Leioa, Spain

## Abstract

Several stem cell sources persist in the adult human body, which opens the doors to both allogeneic and autologous cell therapies. Tooth tissues have proven to be a surprisingly rich and accessible source of neural crest-derived ectomesenchymal stem cells (EMSCs), which may be employed to repair disease-affected oral tissues in advanced regenerative dentistry. Additionally, one area of medicine that demands intensive research on new sources of stem cells is nervous system regeneration, since this constitutes a therapeutic hope for patients affected by highly invalidating conditions such as spinal cord injury, stroke, or neurodegenerative diseases. However, endogenous adult sources of neural stem cells present major drawbacks, such as their scarcity and complicated obtention. In this context, EMSCs from dental tissues emerge as good alternative candidates, since they are preserved in adult human individuals, and retain both high proliferation ability and a neural-like phenotype *in vitro*. In this paper, we discuss some important aspects of tissue regeneration by cell therapy and point out some advantages that EMSCs provide for dental and neural regeneration. We will finally review some of the latest research featuring experimental approaches and benefits of dental stem cell therapy.

## 1. Stem Cells: Neural Differentiation and Regeneration

The human body possesses several sources of stem cells that remain active during adult life. These stem cells are responsible for renewal of differentiated cell pools in the adult organism, and are particularly enriched in tissues that present a high cell turnover, like the hematopoietic bone-marrow and the skin, among others. Stem cell transplantation has been successfully tested at the clinical level to cure several diseases, confirming the big hopes that were placed upon this emerging technique [1, 2]. These accomplishments encourage further research on stem cells for their use in cell therapy. However, several issues need to be fully addressed before the clinical use of stem cells becomes generalized, such as the control of their cellular physiology and their long-term safety [3]. Another important point concerns the discovery of highly accessible tissue sources which may provide a sufficient amount of stem cells for use in autologous cell therapies, or “patient-specific” cell transplants, which is most interesting from the view of graft immunotolerance. Intensive research will be needed to properly evaluate, over the next decades, which will be the true power of cell therapy to repair diseased or damaged tissues on demand. Research on new sources of human stem cells, controlling the differentiation potential of these stem cells and assessing their safety *in vivo*, will be all fundamental to ultimately reach that goal.

The nerve tissue constitutes a particular case where a big social demand for new therapies aimed at its restoration exists. Currently, there is no effective treatment for many devastating diseases and conditions that involve a destruction of nerve tissue, such as brain or spinal cord injury, stroke, Alzheimer's disease, Parkinson's disease, or amyotrophic lateral sclerosis, among others. As the nervous system controls the rest of bodily functions, these neural damages end to be, both physically and psychologically, highly invalidating for the affected patients, representing a huge social burden for both them and their relatives [4, 5]. In spite of the great interest in neural restoration therapies for brain diseases, nerve tissue presents inherent difficulties for its effective regeneration. The central nervous system in an adult human individual contains billions of neurons. Each neuron in turn can receive hundreds of synaptic connections. From the medical point of view, regenerating this complex pattern of neuronal circuitry constitutes an extraordinary challenge. As an additional complication, when nerve tissue is destroyed, glial cells around tend to accumulate and form a fibrous glial scar that prevents growing nerve fibers from penetrating and thus reinnervating the affected area [6].

A major research effort is being currently undertaken to devise new strategies to aid growing nerve fibers to overcome the glial scar. Some approaches relying on cell therapy have been notably successful, including the transplantation of glial cells from the olfactory bulb, which boosts functional recovery in rodent and primate models of spinal cord injury [7, 8]. Nevertheless, if this kind of regenerative approaches was to be translated to the clinic with human patients, efficient methods to provide a sufficient amount of stem cells should be devised. Neural stem cells (NSCs) in the adult human body are found in two main locations: the subgranular zone of the dentate gyrus of the hippocampus and the subventricular zone of the lateral ventricles [9]. Both of them are highly inaccessible areas, only reachable by invasive brain surgery. Furthermore, the amount of neural stem cells that might be obtained from these areas would arguably be very reduced, unless they were harvested from postmortem brain [10], thus discarding autologous cell therapy.

Probably some of the most available and best-characterized human stem cells to date are the multipotent mesenchymal stem cells (MSCs), which can be isolated from the umbilical cord, the bone marrow, and adipose tissue, among others [11]. MSCs have a mesodermal origin, and they are the forming precursors of the majority of connective tissues in the organism, therefore constituting ideal candidates for their use in connective tissue regeneration strategies. Given their availability, abundance, and well-established methods of isolation, the potential of MSCs to generate neural cell phenotypes has been extensively tested. Although this neural differentiation step involves breaching a major cellular differentiation barrier, the one that separates mesoderm from neuroectoderm lineages, this extent has been reported to be possible in numerous studies; for some examples, see [12–17]. However, very serious doubts were raised as to whether the cells obtained in this way correspond indeed to genuine functional neural/glial cells or are just artefactual [18, 19]. Some neural differentiation procedures involve permanent genetic manipulation of MSCs by gene transfection, which would be undesirable from a clinical point of view [20]. More seriously, a lot of the so-reported neural differentiation protocols were subsequently proven to induce merely cytoskeletal shape changes and/or cell death [18, 19, 21]. Additionally, the expression of neural markers, especially when it is only assessed after very short periods (several hours) after application of neural differentiation protocols, appears very weak and unreliable as a sole criterion to define neural transdifferentiation [19]. Therefore, mesodermal MSC transdifferentiation to neural fates has yet to solve important issues before being widely accepted by the scientific community [22, 23]. Finally, up to date, we lack definite evidence that transplanted MSCs whether or not transdifferentiated, are able to form functional synapses with neurons of the host, to integrate, and to effectively replace neural components within a functional brain network.

Although evidence that mesodermal MSCs can indeed transdifferentiate to neurons and integrate in an existing neural network is still to be provided, transplanted MSCs and other stem cells may contribute to nerve tissue regeneration by other mechanisms, such as the secretion of anti-inflammatory cytokines [24, 25], and a big array of growth factors promoting cell survival and angiogenesis [26, 27]. Transplantation of MSC to neural tissue has succeeded in ameliorating the functional outcome in several animal models of brain injury, stroke autoimmune and neurodegenerative diseases [28–33]. Cell fusion and transfer of mitochondria between MSCs and host cells has been demonstrated [34–37], and similar mechanisms could contribute to the healing activity of MSCs *in vivo*. At the present time, we do not certainly know which of the three mechanisms: (i) transdifferentiation, (ii) factor secretion, or (iii) cell fusion, plays the most critical role with regard to the neural function improvement in MSC transplantation experiments [22]. However, these results overall invite to optimism. Whatever the mechanisms underlying it, brain function recovery by stem cell transplantation is possible, and it might not be long before such therapies are broadly available for their use in patients. Moreover, transplanted cells possess a significant advantage over other vehicles for trophic factor delivery, in that they are live dynamics entities capable of interacting with and adapting to their environment [38].

## 2. Neural Crest Stem Cells from Dental and Periodontal Tissues

**Figure 1:**
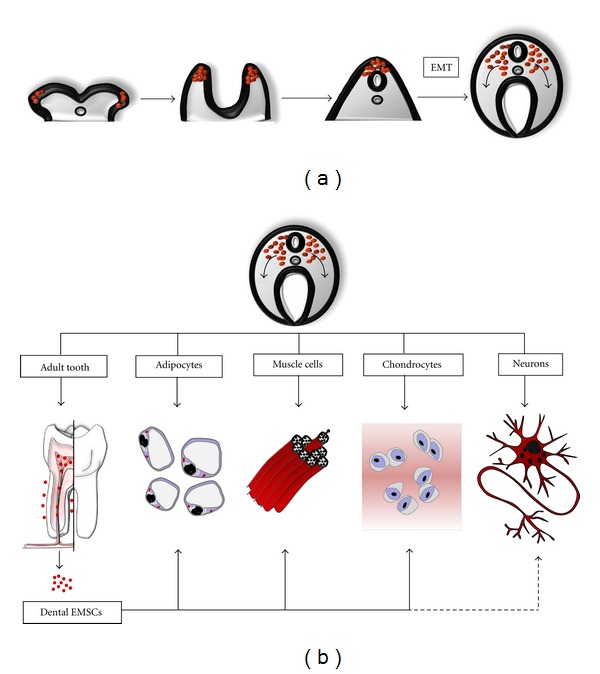
Origin and differentiation potential of dental ectomesenchymal stem cells (EMSCs). (a) Origin of neural crest stem cells (NCSCs). The neural crest arises as a cell population belonging to the fusing edges of the neuroectoderm. After neural tube fusion, neural crest cells undergo an epithelial-mesenchymal transition (EMT), where they transform into EMSCs. EMSCs migrate to generate the majority of craniofacial tissues, including tooth tissues fat, muscle, bone, and cartilage tissues, as well as cranial peripheral ganglia and nerves, among others. (b) EMSCs are retained in the adult dental pulp and periodontal tissues. These cells keep the potential to differentiate to various cell lineages and thus regenerate different dental and connective tissues. Dental EMSCs appear to hold a particularly high neurogenic potential and may also be used to regenerate nerve tissue.

Dental and periodontal tissues constitute a relatively recently discovered source of neural crest stem cells (NCSCs) [39]. The majority of craniofacial connective tissues, including those of the dental pulp and periodontal ligament, are formed by an special type of mesenchymal tissue, derived from the neural crest during embryonic development, thus termed ectomesenchyme [40]. Ectomesenchyme contributes to the generation of craniofacial structures, such as oral muscles, bones, tongue, craniofacial nerves, and teeth, and dental ectomesenchymal stem cells (EMSCs) therefore share a common origin with neural crest cells [41] (Figure 1).

One important feature of dental EMSC is that a substantial amount of these are maintained in the dental pulp and periodontium of both deciduous and permanent teeth [42]. These EMSCs function in the dental pulp *in vivo* to renew populations of dental pulp fibroblasts, and also when needed, to replace injured odontoblastic cells and create a protective layer of reparative dentin [39]. Additionally, EMSCs are also enriched in periodontal tissues, which need a continuous fibroblast cell supply and collagen fiber remodeling to adapt to strong masticatory forces [43].

Dental and periodontal stem cells present a substantial advantage for their use in nerve tissue restoration, in that they present a neural crest phenotype. Contrary to mesoderm-derived MSCs, EMSCs from dental tissues constitutively express neural-progenitor protein markers, even in basal culture conditions [41, 44–46]. This suggests that EMSCs may retain the intrinsic ability to redifferentiate to nerve cells. Due to their common embryonic origin with the peripheral nervous system, it seems reasonable to say that dental EMSCs are one step closer to nerve cells than other stem cells, such as mesodermal MSCs, and thus EMSCs may be more amenable than other stem cells to genuine neural and glial cell differentiation, under the appropriate conditions [41, 47]. This propensity to differentiate to neural lineages is not exclusive to dental EMSCs, and other NCSC types, such as those present in the skin and hair follicles, show similar neural differentiation ability [48, 49]. 

**Figure 2:**
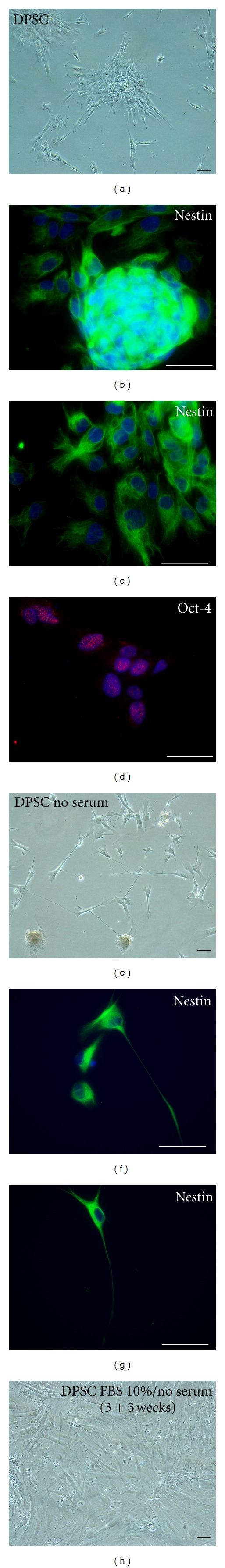
Dental EMSCs express neural differentiation and pluripotency markers and can acquire a prominent neural-like morphology *in vitro*. Dental EMSCs isolated from dental pulp (DPSCs) form clonogenic adherent colonies (a), which present Nestin+ immunoreactivity (b) and from which equally Nestin+ migrating cells spread to eventually bring the culture plate to full confluency (c). Dental EMSCs also express pluripotency markers such as Oct-4, in the absence of any genetic or pharmacological manipulation (d). The cellular morphology and proliferation rates of dental EMSCs vary depending on the presence of FBS in the culture medium. DPSCs proliferate slowly in the absence of serum. Cells cultured without serum are equally Nestin+ but display very variable morphologies, including the appearance of cells with striking neuron-like shape, that show very thin and long cytoplasmic processes, resembling dendrites and axons (f, g). When DPSC are expanded for 3 weeks with 10% FBS, following another 3 weeks of serum deprivation, a sheet of nerve-like tissue is formed (h). Times after seeding: (a) 1 week; (e) 3 weeks; (h) 6 weeks (3 + 3); (b–d; f–g) double merged images of Nestin (green) and Oct-4 (red) immunolabeled cells, with DAPI (blue) introduced as a nuclear counterstain. Scale bars: 50 *μ*m.

The amount of cells that can be obtained from a healthy human molar tooth pulp ranges between 500.000 and 2 million, which may seem quite modest. However, it is estimated that between 0.2% and 0.7% of the cells plated after pulp dissociation represent true colony-forming dental EMSCs, also referred to as dental pulp stem cells (DPSC) [39]. In our experience with these dental pulp cultures, when placed in a culture medium specific for MSC, nonstem cells deadhere and only adherent dental EMSCs remain. These EMSCs rapidly generate Oct-4+/Vimentin+/Nestin+ clonogenic colonies. After 5 days in culture, each of the colonies may show around 40–50 cells on average, and some peripheral cells with fibroblastic migratory shape, showing big lamellipodia, begin to spread apart of the colony cell mass (Figures 2(a)-2(b)). Then, a significant change is observed, notably depending on the absence or presence of fetal bovine serum (FBS) in the culture medium. Cells placed in 10% FBS continue to proliferate at high rate and can be maintained in this condition for very long periods, over 4–6 months, while preserving Oct-4+/Vimentin+/Nestin+ immunoreactivity (Figures 2(c)-2(d)). We estimate that, in the presence of FBS, about 1000 plated EMSCs are capable of bringing a 6-well culture plate area to full confluence (roughly 1 million cells) in the course of merely 2 weeks. Thus, it seems reasonable to say that, although the number of EMSCs that can be obtained from a single tooth piece is indeed small, their high proliferative capacity *in vitro* makes dental tissues very promising alternatives to provide sufficient amounts of EMSCs, even for clinical purposes.

Interestingly, dental EMSCs can also be cultured *in vitro* in the absence of serum for long periods, comparable to those with serum. However, important differences are observed in no-serum conditions. In the absence of FBS, once after the initial steps of colony formation, dental EMSCs cease to proliferate. At this point, some specific cells with a shape surprisingly similar to neuronal cells begin to emerge, displaying long and thin cytoplasmic processes resembling dendrites and axons (Figures 2(e)–2(g)), whereas other cells maintain a fibroblast-like morphology. Overall, in the absence of serum, cell extension throughout the culture plate is limited, restricted to the initially formed stable colonies and a few migrating cells. 6-well culture plates are not brought to confluency even after several months after seeding. To what concerns neural marker expression, the neural progenitor marker Nestin as well as the mature neuron marker *β*-3tubulin is increased under serum-absence conditions, and some cells out of the colonies present a very striking neural-like elongated morphology (Figures 2(f)–2(d)). Therefore, the absence of serum might favor the acquisition of a neural-like phenotype by dental EMSCs, as opposed to a fibroblast-like one. To this date, there is a need to evaluate if these dental EMSCs cultured *in vitro* could represent genuine neuron-like cells. They present strong Nestin+ immunoreactivity, *β*-3tubulin+ immunoreactivity, and surprisingly similar neural-like morphology in the absence of any kind of genetic or pharmacological stimulation of neural differentiation, but further research, including a more detailed information of their transcriptome and electrophysiological assessment of their electrical activity, will be necessary to clarify this. Another important point is to determine if fibroblast-like cells expanded on FBS can be effectively reverted to a neuron-like phenotype after subsequent serum deprivation. Our preliminary results suggest a positive answer to this (Figure 2(h)). This is an important matter, since the presence of xenoproteins on the culture medium might be inappropriate for the use of these cells in human cell therapy [50]. 

## 3. EMSCs Derived from Human Adult Teeth

Different EMSC types can be isolated from dental and periodontal tissues. In this section, we proceed to discuss the main characteristics of the different dental and periodontal stem cell types. Overall, EMSCs derived from adult teeth or those derived from developing teeth can be distinguished.

EMSCs derived from adult teeth would be the most interesting from the clinical viewpoint, because it permits their extraction on demand, whenever they are required, precluding the need of long periods of cryopreservation and storage in cell banks, and therefore saving considerable economic and human resources. Very often, tooth pieces are extracted for other reasons than to obtain stem cells. The most clear example of this constitutes the third molars (or wisdom teeth) of young individuals, which are daily extracted by orthodontic reasons and discarded by thousands as chirurgical waste, in dental clinics worldwide. Therefore, dental tissues may well provide a steady supply of stem cells to be employed in autologous and/or allogeneic transplants in human patients. Finally, third molars have a significant advantage over fully adult erupted teeth, in that they are not completely developed and thus present a more immature phenotype at the time of extraction, which seems to favor the presence of pluripotent-like EMSCs [51]. 

### 3.1. Dental Pulp Stem Cells (DPSCs)

The human adult tooth pulp contains a population of neural crest-derived EMSCs that can be isolated by enzymatic digestion and which forms adherent clonogenic colonies of fibroblast-like cells when cultured *in vitro* ([39, 41]; Figure 1(a)). These EMSCs from dental pulp, termed DPSCs, display a great variability in growth rate and may exhibit a wide range of cell morphologies and tissue marker expression, which appears to reflect their high multilineage differentiation potential to both mesenchymal and nonmesenchymal lineages, characteristic of neural crest stem cells [41, 52]. Notably, DPSCs *in vivo* appear to reside at the perivascular and periodontoblastic compartments within the adult tooth pulp [41, 53], and their cellular phenotype corresponds to pericyte-like smooth-muscle-actin- (SMA-) expressing cells [54]. DPSCs proliferate faster than bone marrow MSCs (BMMSCs) [39, 42]. Finally, and similarly to other MSC types, protocols have been devised that permit long-term cryopreservation of DPSCs without affecting their stemness potential [55]. 

DPSCs have been reported to *in vitro* differentiate to multiple cell lineages, including odontoblasts, chondroblasts, adipocytes, muscle cells, and neurons [44, 56]. Differentiation of DPSC to dentinogenic cell lineages specifically seems to be favored after serial *in vitro* culture passaging [57]. When DPSCs are xenotypically transplanted in immunocompromised mice, combined with mineralized biocompatible hydroxyapatite/tricalcium phosphate (HA/TCP) scaffolding materials, they can generate a complete dentin-pulp complex containing odontoblastic cells. Conversely, BMMSCs in the same conditions give rise to highly vascularized bone-like tissue, containing adipocytes and lamellar bone trabeculae [39, 58]. These different outcomes after *in vivo* transplantation seem to be at least partly related with a differential secretion of paracrine signals by the grafted stem cells, which act upon surrounding host cells to generate specific tissue phenotypes [59, 60].

### 3.2. Dental Pulp Pluripotent Stem Cells (DPPSCs)

Very recently, a new stem population from the human dental pulp of third molars has been isolated and characterized [51, 61]. These cells, termed dental pulp pluripotent stem cells (DPPSCs), may correspond to the aforementioned population of tooth pulp NCSCs [41]. DPPSCs express pluripotency markers such as Oct-4, Lin-28, Sox-2, and Nanog, which are four factors whose induced expression alone is sufficient to revert human-differentiated cells to a pluripotential phenotype [62]. DPPSCs have been shown to differentiate to cells from the three embryonic layers: endoderm, mesoderm, and ectoderm, thus displaying a potency that was widely thought to be exclusive from embryonic stem (ES) cells and induced pluripotent stem (IPS) cells [41, 51]. Similar stem cells with basal pluripotency marker expression have been isolated from the periodontal ligament [63], and our own studies confirm that stem cells derived from the dental pulp of third molars express pluripotency markers, such as Oct-4, which were traditionally associated with pluripotent ES and IPS cells (Figure 2(d)).

### 3.3. Stem Cells from Human Exfoliated Teeth (SHED)

Like adult permanent teeth, pulp tissue of primary exfoliated human teeth (children's milk teeth) also contains an EMSC population. SEHD possess remarkable proliferative capacity, even higher than DPSC [46]. SHED basally express neural markers and can be readily induced to differentiate to neuron-like cells, and transplanted SHED have been shown to survive for a long term and integrate in recipient brain tissues [46]. A basal expression of pluripotency markers has been documented for SHED as well [64]. Therefore, it is quite likely that a population of pluripotent cells, similar to the DPPSCs found in the adult dental pulp, may also be isolated from exfoliated milk teeth. However, the clinical use of SHED in clinical therapy might be hampered by their limited temporary availability and the fact that milk teeth undergo intense root resorption during exfoliation, which may reduce the amount of collected material.

### 3.4. Periodontal Ligament Stem Cells (PDLSCs)

Enzyme-mediated digestion of the PDL also yields a MSC population with clonogenic potential, and similar proliferation rates to adult DPSCs [43], and PDLSCs also display a multilineage differentiation potential [65] and a basal expression of pluripotency markers [63]. When transplanted *in vivo* in the presence of HA/TCP scaffolds, these PDLSCs are able to generate properly arranged cementum and PDL-like structures, including Sharpey's fiber bundles providing cementum attachment [43]. These properties make PDLSCs a good choice for their use in periodontal tissue engineering therapies, possibly in combination with other adjuvant factors such as platelet-derived plasma rich in growth factors, or PRGFs [66]. Moreover, PDLSCs also maintain their stem cell properties after cryopreservation [67].

## 4. EMSCs Derived from Developing Teeth

The formation of the dental pulp and periodontium during tooth development requires the coordinated action of different neural crest stem cells [68]. These stem cells, given their embryonic nature, may not be considered for isolation with clinical purposes, for obvious ethic reasons. They represent nonetheless interesting alternative animal NCSC sources for experimental research in tissue engineering. No pluripotency features have been described yet for EMSCs from developing teeth, although because of their common origin with EMSCs from adult teeth and other nondental NCSC types, it is conceivable that they might share this property as well.

### 4.1. Stem Cells from Apical Papilla (SCAP)

The apical papilla is the tissue which surrounds the apices of developing teeth near Hertwig's epithelial root sheath and which takes part in tooth root formation. An EMSC population presenting a high proliferative capacity can be isolated from there [69, 70]. The apical papilla may also be present in some preerupting little developed wisdom teeth. SCAP can be induced to differentiate to multiple cell lineages and possess a large potential for dental and periodontal repair therapies [71]. SCAP can also be cryopreserved without losing stem cell activity [72].

### 4.2. Stem Cells from Dental Follicle (SCDF)

The dental sac or follicle is the ectomesenchymal embryonic tissue surrounding the tooth germ. This tissue is still present in preerupting impacted wisdom teeth [73]. SCDF can be differentiated *in vitro* to various cell lineages, particularly those involved in the formation of periodontal tissues [74–76].

## 5. Neural Crest Stem Cells and Pluripotency

One outstanding feature reported for dental EMSCs is their apparent pluripotency. As already mentioned, some recents studies suggest that EMSCs from dental and periodontal tissues may possess a superior multilineage differentiation potential, even comparable to that of ES and IPS cells [41, 61]. This may not be so surprising, if we consider the nature of the NCSC phenotype [77]. Already during early embryo development, neural crest cells must undergo a major phenotype switch, to become connective tissue-like migrating cells (i.e. ectomesenchymal cells), out of cells primitively belonging to the epithelial neuroectoderm. This transformation from neuroectoderm to ectomesenchyme, also termed epithelial-mesenchymal transition (EMT), is a clear example of how classic embryonic layer boundaries can be naturally crossed by neural crest cells, and gives evidence of their high multidifferentiation potential [78]. The ability to undergo EMT appears to be important for the emergence of the stem cell phenotype [79], and this is a particularly remarkable and intrinsic feature of adult NCSCs. Consistently, dental EMSCs present upregulated bone morphogenetic protein (BMP) signaling, compared to other mesoderm-derived and phenotypically related stem cells, like BMMSCs [59]. Since transforming growth factor (TGF)/BMP signaling is a major inductor of EMT [80], this may reflect important differences between neural crest-derived stem cells and other adult stem cells, with regard to their multilineage differentiation potential. 

Consistent with the finding of DPPSCs in adult teeth, other NCSCs with similar pluripotency characteristics have been found in additional locations within the adult human body, notably in the skin, and typically associated with hair follicles [81–83]. These skin-derived pluripotent NCSC appear to arise from different locations and correspond to cells that have intense migratory activity [84, 85]. Similarly to DPPSCs, skin-derived NCSCs can be maintained for long periods (months or years) *in vitro*, and they also express neural and pluripotency markers, while retaining the ability to differentiate to different cell lineages, including nerve and glial cells [48, 85]. Notably, skin-derived NCSCs have been successfully used to contribute to functional recovery in animal models of peripheral nerve and spinal cord injury as well as demyelinating disease [86–89]. It remains to be determined whether NCSCs derived from skin and tooth share fully similar characteristics and which might be the conditions that favor the use of one or another type in cell-based regenerative strategies. A substantial advantage of these adult tissue-derived NCSCs for their use in cell therapy is that, contrary to pluripotent ES and IPS cells, they do not appear to form tumors *in vivo* [49, 83, 90].

## 6. Dental and Periodontal Stem Cells in Regenerative Dentistry

Dental EMSCs present an obvious interest for dental tissue bioengineering, because these cells have shown to be able to regenerate various dental and periodontal tissues, after transplantation to immunocompromised animals [91–93]. Affections like dental caries or periodontal disease are very common worldwide, and current procedures to replace missing or degraded dental tissues often rely on synthetic materials, which may not adapt to the patient in the same way as a fully biological graft. In the future, regenerative procedures employing autologous or allogeneic stem cells might become a common dental practice, due to the significant advantages these bioengineered structures would offer, with regard to their better dynamic integration in the oral tissue environment.

Tooth enamel and dentin are degraded in dental caries, whose extreme complication is infection of dental pulp. Nowadays, the most common treatment for such deep caries relies on endodontics, which involves replacement of infected pulp tissue with inert materials. This leaves a devitalized (dead) pulp chamber that results in a much more fragile tooth piece, and may often require placement of a synthetic crown to avoid tooth breakage. Therefore, it is of considerable interest to find bioactive substitutes of pulp refilling materials and in this scenario, dental EMSCs may constitute a very good alternative. Recently, it has been reported that DPSsC, transplanted together with synthetic scaffolds to emptied mice pulp, are able to regenerate pulp chamber tissue and create new layers of dentin *de novo* [94, 95].

Periodontitis is an infective disease that destroys tissues providing tooth anchorage, such as the PDL and surrounding alveolar bone. Severe periodontitis results in a chronic inflammatory condition where risk of tooth loss by detachment is elevated, hence the importance of devising periodontal repair therapies. From the clinical point of view, periodontal repair is a challenging issue, since different tissues (cementum, Sharpey's fibers, PDL, and alveolar bone) need to be restored in a spatially organized way. Notably, positive results of functional periodontal regeneration have been obtained in experimental animals, using stem cell-based strategies [43, 71]. Stem cell therapy thus promises to warrant further research in the field of dental and periodontal repair.

Another research direction of dental and periodontal EMSC biology seeks to exploit the ability of these cells to generate bone [96]. In a dental clinic, boosting alveolar bone generation is of great importance for artificial tooth replacement, given that implants are directly anchored to a predrilled hole in the jaw or maxilla, and these bone areas need to be sufficiently solid to withstand implant screwing and subsequent mastication forces. Regarding this, MSC-based strategies have been proven successful, both experimentally and clinically, to increase bone mass and allow better implant placement and performance [97]. Traditional synthetic implants present a nonnegligible risk of defective integration in the alveolar bone. Another important reason why to switch to autologous cell-based therapies in this context would be to prevent peri-implant disease, which is a far more frequent complication, with prevalence rates estimated of about 8%–14% [98–100]. Experimental evidence reports a significantly better implant tolerance, better bone formation, and higher bone-to-implant contact, when biomaterial-designed implants are placed in combination with PDLSCs or BMMSCs, in animal models of peri-implant defect [101].

The ultimate achievement in regenerative dentistry would be to generate a whole functional replacement tooth, out of cultured and dissociated dental stem cells. Once considered to be chimeric, this has already been successfully accomplished in animal models. Complete and functional mouse teeth can be generated from dissociated dental epithelial and mesenchymal stem cells, properly recombined *in vitro* to reconstitute fully functional bioengineered tooth germs. These tooth germs develop normally to form a well-calcified tooth piece, which integrates well into surrounding tissues, supports masticatory forces, and gets normally innervated [102, 103]. Nevertheless, in spite of these encouraging results, there are several issues to be addressed before this kind of approaches can be translated to dental practice. One of them, not trivial, is patterning of the engineered biotooth germ to achieve optimal dental shape and occlusion [104]. However, the main issue to be solved is the lack of consistent sources of epithelial stem cells with odontogenic potential in the adult human individual to be recombined with mesenchymal stem cells. Despite the inherent difficulty to obtain epithelial odontogenic stem cells (which mostly disappear after tooth eruption), new alternatives are emerging to solve this issue. Recently, positive results have been described using PDL-derived epithelial rests of Mallassez, and oral mucosal epithelial cells [105, 106]. Another interesting alternative to obtain epithelial odontogenic cells would be to derive them from autologous IPS cells [107].

## 7. Dental and Periodontal Stem Cells in Neural Regeneration

Interest in dental stem cell research goes far beyond applications for dentistry. Because of their shared common origin with the nervous system, dental EMSCs may be ideal candidates to generate large pools of neural cells for cellular therapy, in conditions such as ischemic stroke, spinal cord trauma, and neurodegenerative diseases. As already commented before, the properties of dental EMSCs are largely shared by other NCSC types found in other adult human tissues, like the skin and hair follicles. Because of their high accessibility and apparent safety, these adult NCSC types may be regarded as very convenient sources of autologous stem cells for neural regeneration. In this last section, we outline some important aspects with regard to nerve tissue regeneration by cell therapy and discuss the evidence that supports a beneficial effect of dental and periodontal EMSC transplantation for the treatment of neural lesions.

First of all, when it comes to consider the choice of new stem cell sources like dental tissues, size matters. Millions of transplanted cells may be required to repair specific damages, the amount of course depending on the actual lesion volume. Small localized lesions may require fewer transplanted cells, but any suitable stem cell therapy should take the magnitude factor into account. Dental tissues have been partly neglected so far because they constitute a relatively little amount of biological material for the isolation of stem cells, especially when comparing it with large available fat, umbilical cord, or bone marrow tissue sources. However, despite being less abundantly available, dental EMSCs present higher proliferation rates than bone marrow MSCs [39, 46], and their populations can be readily expanded in a few weeks *in vitro*, thus making them a real alternative to classic MSCs. A similar conclusion may be drawn when it comes to evaluate the potential of other related NCSC types, such as those derived from the skin. Large pools of cells can be generated, within 3 months, out of relatively small (2–16 square cm) human skin biopsies [82, 85].

Stem cells derived from adult individuals are particularly interesting with a view to cell therapy of neural disorders, because this would permit to obtain a population of autologous stem cells that would elicit no eventual long-term immune rejection after transplantation. Inflammation is a particularly harmful phenomenon when it refers to nerve tissue. Liquid accummulation characteristic of inflammation increases hydrostatic pressure. Within the central nervous system (CNS), this constitutes a dangerous event which would lead to a rapid collapse of neural function. The CNS has a sophisticated mechanism to avoid liquid filtration across its irrigating blood vessel walls (the blood-brain barrier) and thus prevents a potentially mortal brain edema [108]. The resident macrophages of the central nervous system (the microglia) have evolved to adapt to the specific immune requirements of the CNS as well [109].

For all these reasons, it is very advisable that any eventual clinical trial of cell transplantation to the human CNS attempts to minimize any chance, however little, of immune rejection. Some nonneural crest stem cell types, like MSCs, have been reported to improve neural function in a variety of animal models, which was attributed to an immunosuppressant role, by secretion of a large number of anti-inflammatory cytokines, even when these cells were allogenically transplanted [25]. However, when devising a cell therapy protocol for CNS repair, owing to its extraordinary immune sensitivity, it is highly convenient that the cells of use are autologous, or “patient specific.” Therefore, research on stem cell transplantation to nerve tissue should not only rely on allogeneic cells from compatible human donors, but also and possibly mainly on autologous cells isolated from the own patient. With regard to autologous cell neurotherapy, NCSCs from skin and dental tissues constitute very interesting alternatives, since these can be easily extracted, even in aged patients that may not support well the complicated surgery that is required for the extraction of fat or bone marrow tissue [110]. Moreover, NCSCs from dental and skin tissues may be a far better choice than other stem cell types for neural regeneration [47, 49, 86, 90, 111]. Finally, the immune-regulation benefits described when MSCs are transplanted to the CNS may be comparable, if not better, using autologous dental EMSCs [112, 113].

Dental pulp EMSCs are characterized by a strong expression of neural and glial cell markers, even in basal conditions and in the absence of any pharmacological or genetic manipulation. Moreover, under specific culture conditions, dental EMSCs can be induced to acquire a neural-like morphology, which includes the appearance of very long cytoplasmic processes resembling dendrites and axons (Figure 2). Some studies have already shown that cultured dental EMSCs also show neuron-like electrical activity, characterized by the expression of functional neurotransmitter receptors and the generation of action potentials [47, 114]. Therefore, if properly stimulated, dental EMSCs could constitute a privileged source to obtain neural cells. Furthermore, transplantation of dental EMSCs in experimental animals has shown that these exogenous cells can integrate and survive in the host neural tissue, adopting a neural phenotype according to their specific CNS or PNS location, and even promoting *de novo* neurogenesis [46, 115, 116].

Importantly, the healing potential of stem cell transplantation is known to be based on mechanisms far more complex than a mere engraftment and replacement of damaged cells. Indeed, dental EMSCs have been shown to secrete a wide variety of paracrine factors such as neurotrophins and chemokines [117], which can play critical roles in the survival of neighboring cells, immunomodulation [112, 113], and even axonal guidance [118]. Remarkably, and similar to other MSCs types, dental EMSC have been shown to present strong immunosuppressive properties [112, 113, 119], which constitutes a fundamental factor to understand the achieved therapeutic success of stem cell therapies for nerve injury [120].

The possibility to obtain autologous NCSCs from dental and/or skin tissue and transplant them in the brain or spinal cord of human patients affected by neural damage could yield considerable expectation, since these lesions are usually quite hopeless with regard to spontaneous tissue regeneration and functional recovery. There is a high social demand to devise innovative neural regeneration boosting strategies for these patients, but dramatically at the same time, endogenous sources of human neural stem cells, which could perfectly fit into that picture, are very scarce and nonaccessible. Experimental evidence of dental and skin-derived NCSC engraftment shows damage recovery in animal models of both central and peripheral nervous system injury [49, 86–89, 121, 122]. This clearly opens new doors for hope on the treatment of largely invalidating human neural disorders, like brain and spinal cord trauma, stroke, and neurodegenerative diseases. However, a comprehensive understanding of the healing processes triggered by stem-cell transplant is yet to be achieved. The repair mechanisms are likely to be more complex and diverse than a differentiation to neurons/glia and replacement of damaged cells, and immunomodulatory effects may play an important role. It is becoming clear that dental EMSCs may hold a broad application potential, not only regarding regeneration of oral, dental, and neural tissues, but even for more general conditions requiring a dynamic immune system regulation, like autoimmune diseases.

## 8. Concluding Remarks

Human adult teeth and periodontium retain populations of NCSCs that show characteristics of pluripotency. These stem cells, similar to other NCSCs types in the human body, are highly accessible and offer substantial additional advantages that make them good alternatives for their manipulation and clinical use: they present a high multilineage differentiation potential, high proliferative capacity, they are not oncogenic, and its obtention does not raise ethical concerns. Since the isolation of these cells does not require to make a large tissue biopsy, dental NCSCs are particularly suited for autologous cell therapies. Dental and periodontal stem cells are currently being experimentally tested in various tooth and oral tissue regeneration scenarios. Another great application ground for dental NCSCs is nervous system repair. Both dental and nondental NCSCs express immature neural/glial cell markers and are particularly amenable to neural/glial differentiation. Remarkable positive results of neural regeneration and functional improvement have been obtained in experimental models of brain, spinal cord, and nerve injury therapy, using transplanted dental and non-dental NCSCs. Time and future experiments will tell whether NCSCs from dental tissues become a privileged source of the very much needed neural cells for clinical cell therapy in human patients.
